# Assessing the health burden of vaccine-preventable infections in European adults: challenges and opportunities translated into action

**DOI:** 10.2807/1560-7917.ES.2023.28.48.2300791

**Published:** 2023-11-30

**Authors:** Jade Pattyn, Paolo Bonanni, on behalf of the Adult Immunization Board working group

**Affiliations:** 1 Centre for the Evaluation of Vaccination, Vaccine and Infectious Disease Institute, University of Antwerp, Antwerp, Belgium; 2 Department of Health Sciences, University of Florence, Florence, Italy; 3 Members of the Adult Immunization Board working group are listed under Acknowledgements

**Keywords:** Burden of disease, DALY, Vaccine, adult immunization, life-course immunization

Accurate information on the health burden of vaccine-preventable infections (VPIs) is needed to support evidence-informed vaccine policy recommendations and programmes in Europe. The inaugural technical meeting of the Adult Immunization Board (AIB), held in Antwerp, Belgium, on 20–21 April 2023, convened international experts and was dedicated to the assessment of health burden evidence of VPIs in European adults. 

Presentations and discussion sessions were organised, based on the following pre-defined meeting objectives:

- To provide an overview of currently available vaccines for VPIs in the adult population (≥ 18 years of age)
- To discuss the methodology and challenges in assessing the health burden of adult VPIs
- To evaluate the health burden evidence of selected VPIs to provide a convincing case for strengthening adult vaccination in Europe
- To understand how health burden estimates of adult VPIs shape national vaccination policies and practices and inform public health priorities

This meeting report presents the opportunities and challenges that were identified. Several European initiatives promote health burden of disease (BoD)-harmonised methodologies and/or capacity building collaborations that can be further built upon. Although VPI health burden data are available and are key components in the evidence-informed decision-making processes behind immunisation strategies, data gaps remain, particularly for certain diseases and at-risk populations.

## Overview of currently available vaccines for VPIs in the European adult population

A presentation by Joe Schmitt (University of Cologne, Germany, Global Health Press, Singapore) summarised the current status of adult vaccines and recommendations. There are currently more than 25 VPIs for which adult vaccines are available in Europe. This includes vaccines used in routine adult vaccination programmes (including booster doses of childhood vaccinations), vaccines to catch up on missed child or adolescent vaccinations, travel-related vaccines, occupational activity-related vaccines and other individual risk-based vaccines (e.g. vaccines indicated in pregnancy, for specific medical conditions or lifestyles). Supplement A provides an overview of current adult vaccine landscape in Europe. The list covers licensed vaccines (up to October 2023) in adults.

Vaccine availability, recommendations and reimbursement policies vary over time and between and/or within European countries. Recommended vaccines in each European Union/European Economic Area (EU/EEA) country can be found in the vaccine scheduler on the European Centre for Disease Prevention and Control (ECDC) website (https://vaccine-schedule.ecdc.europa.eu) and on official national public health websites.

## Assessing the health burden of adult VPIs

A set of presentations by several international experts at the meeting (see list with names in the meeting programme) referred to ranking (infectious) diseases in terms of their (potential) burden, which can guide policymakers and help prioritise interventions and use of available resources. Burden of disease is the comparative quantification of disease impact on one or more domains of life, including health, socioeconomic and psychosocial well-being. Health BoD may be evaluated using multiple measures, ranging from case numbers to indicators of disease severity (e.g. disease duration, reduction of quality of life) and death (e.g. number of expected life-years). Depending on the selected measure, the ranking of diseases will be considerably different.

To obtain comparative BoD metrics, summary measures of population health (SMPH) that integrate multiple measures, such as disability-adjusted life years (DALYs), can be used. Transparency and harmonisation of SMPH are required as different methodological choices can be applied, affecting the comparability and interpretation of results.

Periklis Charalampous (Department of Public Health, Erasmus MC University Medical Center, Rotterdam, the Netherlands) presented his systematic review that analysed the methodological design choices of burden of infectious disease studies in the EU and the United Kingdom (UK) [1]. Studies estimating burden in terms of years of life lost (YLL), years lived with disability (YLD) and/or DALY using their own national or sub-national data were included. Overall, 105 studies were identified with publication dates between 2000 and 2022, including 25 studies elaborating on the burden of VPIs. In line with other reviews, DALY methodological choices varied across European-based burden of infectious disease studies.

Health BoD evaluation initiatives, ranging from dedicated research activities and formation of networks to developing technical guidance and BoD data sources, exist at (sub)national, regional and global levels (e.g. Global Disease Burden Study, https://www.healthdata.org/research-analysis/gbd). European-level BoD initiatives, with a particular interest in burden of VPIs, include the Burden of Communicable Diseases in Europe (BCoDE, https://www.ecdc.europa.eu/en/publications-data/toolkit-application-calculate-dalys), the European Burden of Disease Network (https://www.burden-eu.net), and Vaccines and InfecTious Diseases in the Ageing population (VITAL, https://vital-imi.eu). These initiatives strive to improve BoD evidence, either by providing data, harmonising methods, and/or strengthening collaborations among all stakeholders involved.

In addition, during the meeting, the point was raised by Anindya Bose (Department of Immunization, Vaccines and Biologicals (IVB), World Health Organization (WHO), Geneva) that the WHO Immunization Agenda 2030 recommends building and strengthening comprehensive VPI surveillance systems as an essential component to national public health surveillance in order to replace the historically pathogen-focused programmes. This would allow for resource sharing and capacity building, ultimately improving the data provided for BoD estimates.

## Epidemiology and health burden of selected VPIs

To further explore the availability and the quality of the data that are used to estimate the health BoD of VPIs, the meeting participants reviewed the epidemiology and health BoD of pre-selected adult VPIs, in specific situations (e.g. pandemic) and risk groups (e.g. travellers, immunocompromised, older and younger adults). Experiences from the different VPI fields were shared and key points are reported here.

### Burden of a VPI in a pandemic situation: the example of COVID-19 

Sara Monteiro Pires (European Burden of Disease Network National Food Institute, Denmark) discussed VPI burden in the context of the COVID-19 pandemic, emphasising that conducting BoD studies during this period presented multiple challenges, including the lack of data on the full spectrum of health effects of the new emerging virus. Nevertheless, it was an unprecedented opportunity regarding the abundance of surveillance data available and the chance to apply a standard methodology across countries. COVID-19 BoD studies applying the protocol of the European Burden of Disease Network have been carried out in 10 EU countries and Australia [2]. Burden of disease estimates have varied widely, ranging from 32 to nearly 2000 DALYs per 100,000 inhabitants. Differences may reflect variations in population age structure or pandemic response but also data collection, data management, degree of ascertainment of the true incidence of infection, and case or mortality definitions. Moreover, for a same country, results obtained based on aggregated datasets (e.g. ECDC or WHO datasets) may differ from those obtained from more detailed national datasets. For COVID-19, results based on national estimates have tended to yield higher BoD estimates than those based on European aggregated data [3].

### Burden of VPIs in specific risk groups: the example of travellers and immunocompromised persons

Robert Steffen (University of Zurich, Switzerland) and Per Ljungman (Karolinska University Hospital, Sweden) examined the effect of VPIs in risk groups. In adult travellers, despite high-exposure risks, data on burden of VPIs are particularly limited, with the quality of evidence rated low to moderate [4]. For immunocompromised persons, although data on the epidemiology and burden of VPIs are available, the evidence is not always granular enough for this extremely heterogenous group, which includes individuals with different causes and levels of immunosuppression. Faced with these gaps in data, obtaining composite measures such as DALY or other SMPH is currently challenging, and the selection of the most representative individual outcome measures to estimate burden is key.

### Burden of VPIs in older adults: the examples of respiratory syncytial virus and herpes zoster

Presentations by Stefania Maggi (National Research Council-NI-Aging Branch, Italy), Xiao Li (Centre for Health Economics Research and Modelling Infectious Diseases, Belgium), Angela Bechini (University of Florence, Italy), and Hester E de Melker (National Institute for Public Health and the Environment (RIVM), the Netherlands) discussed the important gaps in VPI burden data in the older adults (> 60 years). They find that the lack of data granularity is also applicable to older adults, a heterogenous group with a broad age range, a heterogeneous risk profile and frailty spectrum. An example has been the lack of a comprehensive understanding of respiratory syncytial virus (RSV) burden in older adults, presented by Xiao Li. Challenges for the assessment of RSV BoD include under-ascertainment (e.g. atypical presentations, limited laboratory testing, differences in disease coding, lack of test sensitivity) and lack of harmonised study methods. The arrival of new RSV vaccines for older adults has prompted several European initiatives to address these issues and reduce knowledge gaps (e.g. REspiratory Syncytial virus Consortium in Europe (RESCUE) [5]). Conversely, BoD of herpes zoster in older adults is well described, yet only 10 EU/EEA countries have implemented a vaccine programme for older adults according to the ECDC vaccine tracker, and not all offer national health system funding.

### Burden of VPIs in younger adults: the example of human papillomavirus 

Paolo Bonanni (University of Florence) discussed the implications of VPIs in younger adults, highlighting that human papillomavirus infection and disease burden data are mostly derived from the Global Cancer Observatory reports [6]. These show major health inequalities within Europe. Importantly, the methods and quality of the burden data collected in these reports vary widely between countries, correlating with the underlying surveillance system methodology and capacities. Moreover, despite proven impact of HPV vaccination on BoD both at population and individual levels [7], absence of HPV vaccination programmes and/or poor HPV vaccine coverage could be improved in several European countries.

## How health burden estimates of adult VPIs shape national vaccination policies

To shape and strengthen adult vaccination strategies, health burden of VPIs must be effectively translated into action, with tailored communication of results to its key stakeholders, including the National Immunization Technical Advisory Groups (NITAGs) and health policymakers. A set of presentations on this topic were presented by several international experts at the meeting (see list with names in the meeting programme).

Representatives from four European NITAGs (Finland, Czechia, Germany, Greece) described their respective recommendation processes during the meeting. Invariably, all four NITAGs use BoD to guide vaccine recommendations and evaluate their impact. However, there are differences in their respective decision-making frameworks and inequalities with regard to each country’s capacity to produce local high-quality BoD, cost-effectiveness data or both. The challenges and opportunities for the generation and translation of data on the VPI health burden into action identified during the meeting are summarised in Supplement B.

While BoD data are central for decision-making, adult vaccine recommendations are driven by various other factors, including surveillance data, economic factors, public health security, insurance strategies, ageing policies, vaccination strategies and presence of adult immunisation experts within NITAGs [8].

Geographical disparities are found at all levels of adult immunisation in Europe. Differences exist in surveillance strategies, recommendations, decision-making processes, vaccine implementation strategies and funding, and ultimately vaccine uptake. Moreover, lack of clarity around the rationale for inter-country differences in vaccine recommendations may, in turn, affect vaccine confidence. Therefore, despite intrinsic differences, standardisation of approaches and/or improved communication strategies would be highly valuable and could increase public confidence in local vaccine recommendations.

## Conclusion

Chloé Wyndham-Thomas (P95, AIB rapporteur) summarised the discussions during the meeting, pointing out that high-quality BoD data are key for evidence-informed vaccine policymaking. Ideally, SMPH such as DALYs, which consider multiple factors, are preferred. However, these measures require extensive data collection and resources. Several European initiatives promote harmonised health BoD methodologies and/or capacity building collaborations that can be further built upon. Nevertheless, BoD estimates will only be as good as the data inserted into the models. As such, efforts to harmonise and improve the quality of Europe’s underlying VPI surveillance are equally fundamental. Political support will be needed to move forward, and raising awareness on the full potential of independent health BoD data are required.

The improvement of the quality of data sources and BoD estimates is a continuous process. Meanwhile, in the absence of national high-quality BoD data should never hinder the endorsement of immunisation/vaccines. Although country-specific data are best to underpin national recommendations, BoD evidence from similar demographic or socioeconomic conditions and/or mathematical models may be used as proxy evidence [9]. These alternative data sources should be given significant importance, outweighing the absence of certain data and erasing any justification for postponement.

Importantly, even high-quality BoD estimates will have limitations that should be considered when interpreting results. The comprehensive interpretation of BoD estimates always requires strong knowledge of the methodology, the study setting and the surveillance systems behind the data sources. An illustrative example is the implementation of an improved or broader surveillance system after the initiation of a vaccine programme. This may lead to an increase in BoD related to better case detection, masking the true impact of the vaccine introduction.

Historically, vaccine programmes have focused on preventing disease in children. Now, the paradigm is changing, and vaccine programmes are evolving into lifelong strategies, with vaccines specifically indicated for adults. Nonetheless, the availability and quality of adult BoD and vaccine coverage data varies by pathogen (e.g. RSV) and adult sub-population (e.g. immunocompromised persons and travellers), with remaining gaps and major geographical differences.

To strengthen adult vaccination in Europe, BoD must be effectively translated into action. Communication tailored to the different stakeholders is needed, with improved delivery of results and connection to the political agenda. A comprehensive strategy, using both BoD and other drivers of decision-making is necessary if we are to provide a convincing case for adult vaccination.

**Key conclusions from the meeting:**

• Health BoD data are essential for evidence-informed vaccine policy decision-making and for the monitoring of interventions (e.g. vaccination programmes).

• Harmonisation of BoD methods is necessary to allow comparability and interpretation of results across studies.

• Health burden studies are resource demanding and require extensive high-quality data: efforts to improve Europe’s infectious disease surveillance and collaborations offering both capacity building and cost-sharing should be further promoted.

• Geographical differences and inequalities are found at all levels of adult immunisation in Europe (e.g. in surveillance strategies and data collection, data quality, vaccine recommendations and uptake) and should be considered when interpreting BoD results.

• The communication of VPI health BoD should be tailored to each stakeholder (e.g. ministry of health, NITAG, healthcare providers, general population) and combined with other drivers of political decisions (e.g. health economics) to ensure effective translation into vaccine policy.

## Supplementary Data

270000 Supplement
