# Supplementary material for: Assessing the health burden of vaccine-preventable infections in European adults: challenges and opportunities translated into action
Source: Euro Surveill. 2023 Nov 30;28(48):2300791. doi: 10.2807/1560-7917.ES.2023.28.48.2300791 (PMC10690864; doi:10.2807/1560-7917.ES.2023.28.48.2300791)
Supplement: Supplement [file 23-00791_PATTYN_Supplement.pdf]

This supplementary material is hosted by Eurosurveillance as supporting information alongside the article “Assessing the health burden of vaccine-preventable infections in European adults: challenges and opportunities translated into action” on behalf of the authors who remain responsible for the accuracy and appropriateness of the content. The same standards for ethics, copyright, attributions and permissions as for the article apply. Eurosurveillance is not responsible for the maintenance of any links or email addresses provided therein.

Supplement 1: Overview of current adult vaccine landscape in Europe (>18-years-old). The list covers adult vaccines up to October 2023.

**Overview of current adult vaccines used in Europe (>18-years-old)**					
**Vaccine**	**(Age-dependent) routine vaccines**	**Catch-up missed child/ adolescence vaccines**	**Individual risk-based vaccines (e.g pregnancy, lifestyle, medical condition, etc.)**	**Travel-related vaccines**	**Occupational activity-related vaccines (e.g. HCP, CCW, people who work with animals, etc.)****
**Anthrax**			x		x
**Cholera**				x	
**COVID-19**	x		x	x	x
**Dengue**				x	
**Ebola**					x
**Hepatitis A**		x*	x	x	x
**Hepatitis B**		x	x	x	x
**Hib**			x		
**HPV**		x	x		
**HZ**	x		x		
**S. Influenza**	x		x	x	x
**Pneumococcus**	x	x	x		
**JE**				x	
**MenACWY**		x	x	x	
**MenB**		x	x	x	
**MMR(V)**		x			
**Mpox**			x		
**Polio**		x		x	
**Rabies**				x	x
**RSV**	x		x		
**TB**			x		x

**TBE**	x	x*	x	x	x
**Tdap**	x	x	x		
**Typhoid fever**				x	
**Yellow fever**				x	

Hepatitis E and Q-fever vaccines are not included, as these vaccines are not licensed in Europe. Abbreviations: CCW, child care workers; HCP, health care providers; Hib, Haemophilus influenzae b; HPV, Human Papilloma Virus; HZ: Herpes Zoster; S. Influenza: Seasonal Influenza; JE, Japanese Encephalitis; Men, Meningococcal; MMR(V): Measles Mumps Rubella Varicella; RSV, Respiratory Syncytial Virus; TB, tuberculosis; TBE, Tick-Borne Encephalitis; Tdap, Tetanus diphtheria acellular Pertussis; * Depends whether there is universal childhood vaccination at (sub)national level. ** All vaccines can be given to lab workers working with these pathogens

This supplementary material is hosted by Eurosurveillance as supporting information alongside the article "Assessing the health burden of vaccine-preventable infections in European adults: challenges and opportunities translated into action" on behalf of the authors who remain responsible for the accuracy and appropriateness of the content. The same standards for ethics, copyright, attributions and permissions as for the article apply. Eurosurveillance is not responsible for the maintenance of any links or email addresses provided therein.

Supplement 2 Health burden of VPI and diseases: challenges, opportunities, and improvement strategies identified by the AIB

BoD Opportunities	BoD Challenges	Improvement strategies and ongoing initiatives
**Ranking diseases in terms of their burden can guide national policymakers and help prioritise and evaluate interventions (e.g. vaccination programmes).**<br>**Collection of high-quality BoD data can increase our actionable knowledge.**	**Lack of standardisation** of methods in BoD studies limits **comparability and interpretation** of results.	**Harmonisation** of methodologies and use of **standard protocols and reporting guidelines.** Standardisation initiatives are ongoing by the European Network of Burden of Disease.
**Summary measures of population health such as DALYs integrate multiple outcome measures.**	**Extensive data requirements and important resources needed** (funding and capacity).<br>Current BOD estimates have wide uncertainty intervals.<br>**Not all BoD can be accounted for** (e.g., loss of independence in older adults) and focus on health dimension has its limitations.	**Collaborative and comprehensive platforms** to build on existing initiatives, with capacity building and resource sharing, and prevention of parallel and overlapping initiatives.<br>Collaborative platforms and initiatives include BCoDE (currently inactive) and VITAL.
**BoD indicators can be useful for monitoring within and across-country public health in both non-pandemic and pandemic situations.**	**Differences across European countries** (data sources, data collection, case definitions, geographical and socio-economical settings, healthcare organisation and clinical practices) make comparable burden estimates challenging to generate.	**European strategies**, albeit adapted to the country's reality, to standardise and harmonise data collection and analysis methods. Surveillance standards have been published by the WHO and harmonised protocols and case definitions are proposed by the ECDC.
**BoD estimates are used to generate VPI epidemiology, vaccine effectiveness and vaccine cost-effectiveness data that inform NITAGs and the decision-making process and ultimately vaccine market.**	**Sub-optimal registration records and surveillance of infectious diseases**, including underreporting, under-ascertainment and insufficient data quality and processing, exists in many countries.<br>**BoD data gaps for key pathogen** (e.g., RSV) and **risk groups** (e.g., older adults, IC, travellers).<br>**Lack of vaccine coverage data and/or targets** in vaccine programmes.	**Improve routine registration records and surveillance of infectious diseases**. Develop studies to correct for underreporting of targeted VPIs.<br>Raise awareness among policymakers of the potential of BoD studies and emphasise pandemic preparedness.<br>Leverage COVID-19 capacities.<br>Investment in data linking (e.g. EHDS).

**To make a convincing case for adult vaccination, BoD results are to be effectively translated into policymaking.**	BoD data are **complex to communicate** to a broad array of stakeholders.<br>BoD are **not the only driver in the decision-making process** nor vaccine recommendation.	**Improve result delivery to the political pathway** and connect with political agenda.<br>Adapt BoD translation and communication to the data user (e.g., Ministry, NITAGs). Integrate with other policymaking drivers (e.g., Health economics).

Abbreviations: BoD, Burden of Disease; BCoDE, Burden of Communicable Diseases in Europe; DALY, Disability-Adjusted Life Year; ECDC, European Centre for Disease Control; EHDS: European health data space; IC, immunocompromised. NITAGs, National Immunization Technical Advisory Group; RSV, Respiratory Syncytial Virus; SMPH, Summary Measures of Population Health; VITAL, Vaccines and InfecTious Diseases in the Ageing population; VPI, vaccine-preventable infections; WHO, World Health Organization
